# The legally protected fungi and lichens in the EU

**DOI:** 10.3897/imafungus.17.202399

**Published:** 2026-08-24

**Authors:** Daniel Janowski

**Affiliations:** 1 Institute of Dendrology, Polish Academy of Sciences, Parkowa 5, PL-62-035 Kórnik, Poland Institute of Dendrology, Polish Academy of Sciences Kórnik Poland

**Keywords:** Biodiversity, European Union, fungi, IUCN Red List, lichens, protected species

## Abstract

Despite decades of recommendations from the scientific community, fungi have yet to be included in any international legal instruments that regulate wildlife species conservation in the European Union (EU), such as the Bern Convention. However, several EU member states include lists of protected non-lichenized and lichenized fungal species in their national legislation. Most of these legal documents are available only in local languages and are dispersed across national legal databases. To increase the visibility of fungi protected across the EU and facilitate comparisons of these regulations, the first consolidated list of 884 non-lichenized and 787 lichenized fungal taxa protected under the national laws of 18 EU member states is presented. The shortcomings of the current framework for protecting fungal species are highlighted, the distinction between non-binding Red Lists and legally binding species protection is clarified, and improvements to enhance the effectiveness of fungal conservation laws in the EU and beyond are proposed.

## Introduction

Legal protection of vulnerable species is a key legislative instrument for preventing biodiversity loss. The main documents listing species under legal protection in the European Union (EU) include the Bern Convention (Council of Europe 1979), the Habitats Directive (European Union 1992), and the Birds Directive (European Union 2009). These documents include appendices listing plant and animal species; however, despite decades of calls from the scientific community (Arnolds 2001; Dahlberg and Croneborg 2006; Dahlberg et al. 2010; Heilmann-Clausen et al. 2015), equally conspicuous and ecologically important fungi are largely absent, with the exception of *Cladonia* sect. *Cladina*, a group of lichenized fungi listed under plants in Annex V of the Habitats Directive.

Fungi are crucial to terrestrial ecosystems. They drive nutrient cycling, support vegetation through symbiotic associations, and build ecosystem resilience (Niskanen et al. 2023). Despite the ecological importance of fungi, the recognition and implementation of fungal conservation have lagged behind those of plant and animal conservation (Heilmann-Clausen et al. 2015; May et al. 2018). For a long time, fungi were grossly underrepresented in global conservation assessments: in 2018, only 56 fungal species were assessed for the global IUCN Red List, compared with 25,452 plant and 68,054 animal species (Ainsworth et al. 2018). This number has grown rapidly in recent years, reaching more than 1,300 assessed species in 2026 (IUCN 2026), yet it remains low, given that fungi comprise ca. 20% of all eukaryotic species (Niskanen et al. 2023). Of the fungi assessed by the IUCN, nearly 50% are globally threatened (Vulnerable, Endangered, or Critically Endangered; Mueller et al. 2022).

Currently, Red Lists of fungi have been compiled in most EU member states, listing species of conservation concern; however, Red Lists are non-binding advisory tools and do not confer legal protection (Glasnovič et al. 2024; Lin et al. 2025). Although distinct, Red Lists and legal species protection are often confused in policy discussions (Lin et al. 2025). Although there is currently no EU-level list of protected fungi, several member states have independently included fungi on national lists of protected species (Floccia et al. 2024). These lists are scattered across national legal databases; most are available only in their respective national languages, do not reflect taxonomic revisions, and contain many outdated species names. The fragmented nature of national legislation, combined with the absence of a coordinated EU-level list of protected fungal species, complicates efforts to compare conservation priorities among countries and develop consistent approaches to fungal protection across borders.

This work presents the first comprehensive list of non-lichenized and lichenized fungal species protected under national law across EU member states. For the convenience of researchers, legislators, and other stakeholders, the lists are standardized using current fungal taxonomy, with synonymous names used in legislation reported in a separate column. For all applicable taxa, the global IUCN Red List status (IUCN 2026) and whether a species was previously identified among the 33 priority threatened fungi in Europe (Dahlberg and Croneborg 2006) are reported. The compiled data are summarized, and opportunities to improve legal frameworks for fungal conservation within the EU are discussed.

## Materials and methods

### Procuring national protected fungal species lists

National legislation listing legally protected non-lichenized and lichenized fungal species was identified for all EU member states using a stepwise verification approach.

First, a survey-based report by the Joint Network for Wild Fungi (JoNeF) within the EU Network for the Implementation and Enforcement of Environmental Law (Floccia et al. 2024) was consulted. The JoNeF survey compiled questionnaire responses from European countries on whether national legislation for fungal conservation existed and, in several cases, identified the titles of relevant legal acts. Using the JoNeF report as a starting point, the full consolidated texts of all identified legal acts were independently retrieved from official national legal repositories (e.g., government gazette databases and official legal portals), and the species lists were extracted from these documents.

Second, for countries that reported no fungal protection, provided incomplete responses, or did not respond to the JoNeF survey, official national law databases and government legal portals were consulted directly using keyword-based searches combining translations of “fungi,” “mushrooms,” and “lichens” in the respective national languages with terms related to species protection. These searches were further supported by broader internet searches in both English and the local languages.

Finally, for countries where no legislation specific to fungi was identified or where such legislation did not include any lichen taxa, national lists of protected plant species were consulted to identify any fungi or lichens listed therein.

All retrieved regional legal acts were excluded from the subsequent analysis, which focused on national-level legislation. Four EU countries that legislate fungal species protection only at the subnational level were excluded: France, Italy, and Spain include no fungi or lichens on their national lists of protected species, although some regions have introduced local legislation protecting selected fungal or lichen taxa; in Austria, species protection is delegated entirely to the provincial level. In these four excluded countries, no fungal species are protected nationwide. All legal documents were first retrieved in December 2025 and later verified for amendments up to May 2026. The legal documents listing fungi and lichens protected in EU countries are presented in Table 1.

**Table 1.** National legal documents listing protected non-lichenized and lichenized fungal taxa in EU member states.

**Country**	**English title**	**Corrected no. of listed taxa (non-lichenized/lichenized)**	**Last amendment**	**Reference**
Bulgaria	Biological Diversity Act	10/0	2024	Bulgaria 2002
Croatia	Ordinance on Strictly Protected Species	311/35	2016	Croatia 2013
Croatia	Ordinance on the Collection of Native Wild Species	109/3	-	Croatia 2017
Cyprus	Protection and Management of Nature and Wildlife Law	0/1*	2015	Cyprus 2003
Czech Republic	Decree implementing certain provisions of Act No. 114/1992 Sb., on the protection of nature and the landscape	46/0	2018	Czech Republic 1992
Estonia	List of Species under Protection Categories I and II	36/33	2025	Estonia 2004a
Estonia	List of Species under Protection Category III	10/18	2014	Estonia 2004b
Finland	Government Decree on Nature Conservation	247/472	-	Finland 2023
Germany	Federal Species Protection Ordinance	18/8	2013	Germany 2005
Hungary	Decree on protected and strictly protected plant and animal species, strictly protected caves, and species of conservation significance in the European Community	59/18	2025	Hungary 2001
Ireland	Flora (Protection) Order	0/1*	-	Ireland 2022
Latvia	Regulations on Strictly Protected and Restricted-use Strictly Protected Species	62/60	2022	Latvia 2000
Lithuania	List of Protected Fauna, Flora and Fungi Species of the Republic of Lithuania	74/44	2026	Lithuania 2003
Luxembourg	Grand-Ducal Regulation of 8 January 2010 on the full and partial protection of certain species of wild flora	55/117	-	Luxembourg 2010
Malta	Flora, Fauna and Natural Habitats Protection Regulations	3/2	2025	Malta 2006
Poland	Regulation of the Minister of the Environment on the protection of fungal species	114/199	-	Poland 2014
Romania	Emergency Ordinance No. 57 of 20 June 2007 on the regime of protected natural areas, the conservation of natural habitats, and wild flora and fauna	0/1*	2025	Romania 2007
Slovakia	Regulation of the Ministry of the Environment implementing the Nature and Landscape Protection Act	88/44	2024	Slovakia 2021
Slovenia	Decree on the Protection of Wild Plants	0/1*	2014	Slovenia 2004
Slovenia	Decree on the Protection of Wild Fungi	41/0	-	Slovenia 2011
Sweden	Swedish Species Protection Ordinance	5/8	2022	Sweden 2007

*Lichenized fungi listed under plants.

### Preparing consolidated lists

From each identified legal document, all listed taxa were extracted and classified as non-lichenized fungi or lichenized fungi. These were compiled into two separate working lists. Where legislation specified multiple protection levels, the strictest protection was marked in the consolidated lists as “A,” with successively lower levels marked as “B” and “C.” These labels were used only to preserve the relative hierarchy of protection within each country; the protection levels are not directly comparable across member states.

Species names were standardized against MycoBank (www.mycobank.org; accessed January 2026; Robert et al. 2013). Where the currently accepted name differed from the name used in legislation, the accepted name was adopted, and the legislative name was retained in a separate column for reference. Orthographic errors in species names listed in the legal texts were corrected during standardization.

After standardization, duplicate species entries across countries were merged, and country-specific protection levels were recorded in dedicated columns. Taxonomic family affiliations were recorded for each species based on MycoBank. Each taxon was then cross-referenced with the global IUCN Red List (IUCN 2026) to determine its global conservation status, where available. In addition, fungal species on the list of 33 threatened fungi in Europe (Dahlberg and Croneborg 2006) were indicated.

### Statistical analyses

To examine patterns across national protection lists, multivariate analyses were performed on presence–absence matrices derived from the consolidated lists. Species composition dissimilarity between countries was quantified using the Sørensen index, calculated separately for non-lichenized fungi and lichens. Dissimilarity was visualized using hierarchical clustering dendrograms constructed with the unweighted pair group method with arithmetic mean. Mantel tests (999 permutations, Spearman correlation) were used to assess the relationship between species composition dissimilarity and geographic distance between country centroids. Nestedness was evaluated using NODF (nestedness metric based on overlap and decreasing fill), with significance tested using a row-shuffle null model (999 permutations) that preserved country richness while randomizing species identities. To identify compositional biases, chi-square tests of independence were used to compare family composition across countries, and adjusted standardized residuals were calculated to identify countries that significantly deviated from the expected proportions; only countries listing more than 20 taxa were included in this analysis. Taxa protected in only a single country (singletons) were quantified, and differences in global IUCN assessment coverage and family composition between singleton and shared taxa were tested using Fisher’s exact test with odds ratios. All analyses were performed using Python 3.10.12, with Claude Sonnet 4.6 used to assist with script preparation.

## Results

Consolidated lists of legally protected non-lichenized and lichenized fungal species across EU member states are provided in Suppl. material 1: tables S1, S2, respectively.

**Figure 1.**
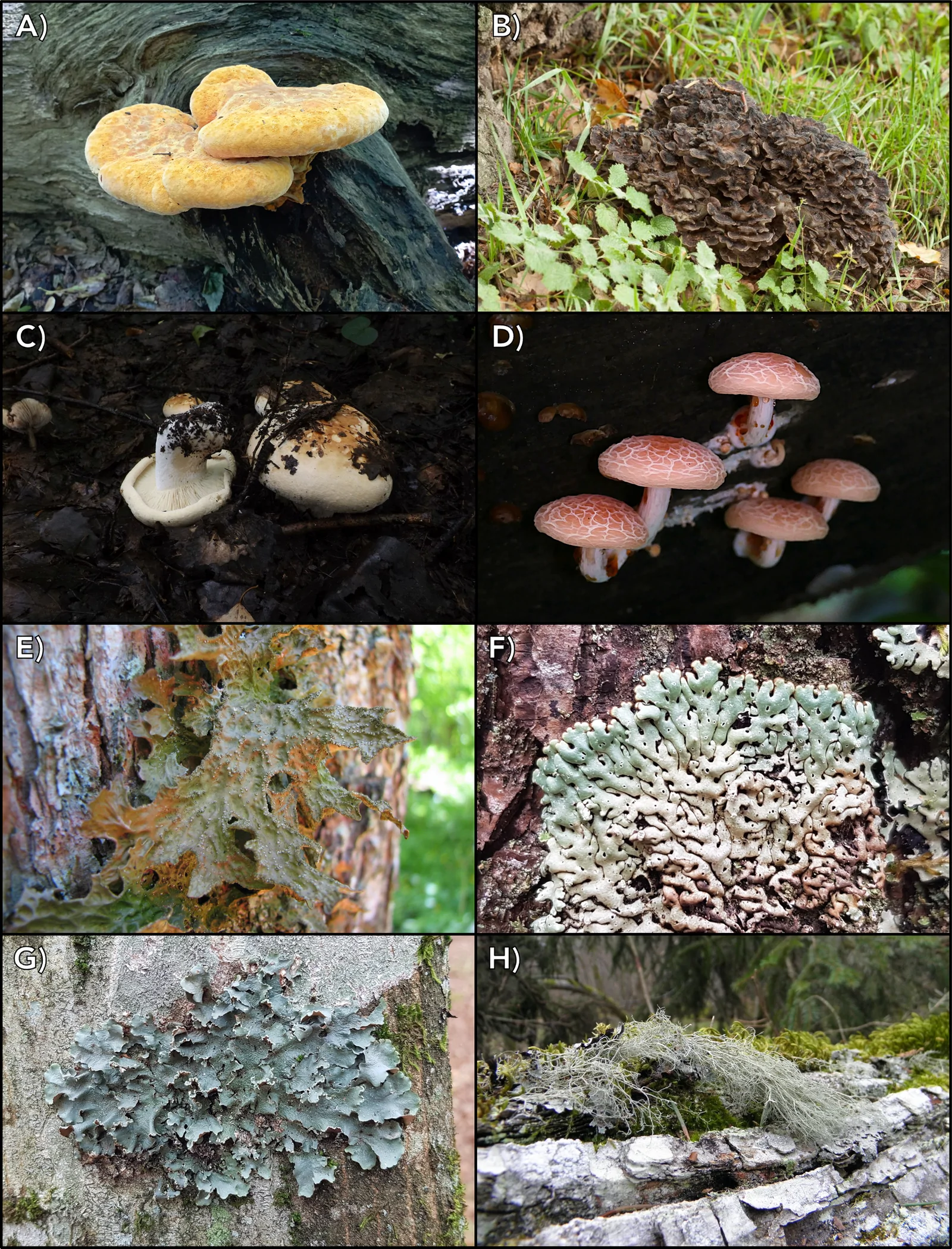
Non-lichenized (**A–D**) and lichenized (**E–H**) fungi protected in the highest number of EU member states. **A**. *Hapalopilus croceus*—protected in HR (Croatia), EE (Estonia), FI (Finland), HU (Hungary), LV (Latvia), LT (Lithuania), PL (Poland), SK (Slovakia), and SE (Sweden); photo by A. Szczepkowski. **B**. *Grifola frondosa*—HR, EE, HU, LV, LT, PL, and SI (Slovenia); photo by K. Kujawa. **C**. *Leucopaxillus compactus*—BG (Bulgaria), HR, EE, FI, HU, LT, and PL; photo by M. Gryc. **D**. *Rhodotus palmatus*—CZ (Czech Republic), EE, HU, LT, LV, PL, and SK; photo by A. Hreczka. **E**. *Lobaria pulmonaria*—HR, EE, DE (Germany), HU, LV, LT, LU (Luxembourg), PL, and SK; photo by R. Szymczyk. **F**. *Menegazzia terebrata*—HR, EE, FI, LV, LT, PL, and SK; photo by R. Szymczyk. **G**. *Cetrelia olivetorum*—EE, FI, LV, LT, LU, PL, and SE; photo by R. Szymczyk. **H**. *Ramalina thrausta*—EE, FI, LV, LT, PL, and SK; photo by A. Bohdan.

In total, 884 non-lichenized and 787 lichenized fungal taxa are legally protected across EU member states. Protected non-lichenized fungi span 146 families, with the most frequently listed families being *Boletaceae* (52 species), *Agaricaceae* (46), and *Russulaceae* (40); together, these three families represent 15.6% of the protected taxa. Lichenized fungi are represented by 94 families, dominated by *Parmeliaceae* (152 species), *Verrucariaceae* (91), and *Ramalinaceae* (46), with these three families comprising 36.7% of the protected lichens. The most widely protected non-lichenized and lichenized fungal species are shown in Fig. 1.

**Figure 2.**
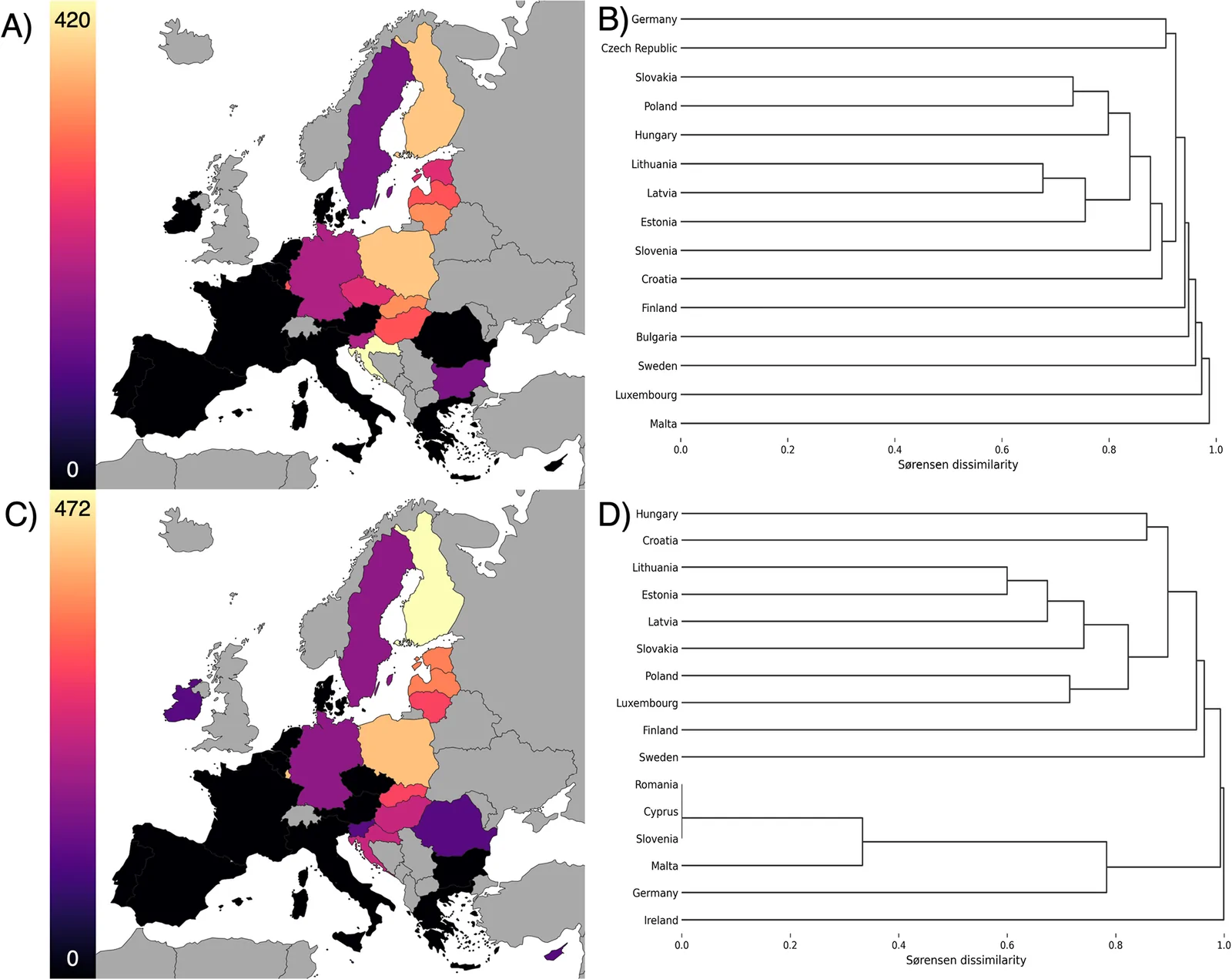
Comparison of national protection lists of fungi across EU member states. Countries with no protected taxa in the respective group are omitted from the dendrograms. © EuroGeographics for the administrative boundaries. **A**. Number of non-lichenized fungal taxa legally protected in each EU member state. **B**. Hierarchical clustering of EU member states based on the Sørensen dissimilarity of their lists of legally protected non-lichenized fungal taxa. **C**. Number of lichenized fungal taxa legally protected in each EU member state. **D**. Hierarchical clustering of EU member states based on the Sørensen dissimilarity of their lists of legally protected lichenized fungal taxa.

Of the 27 EU member states, 15 provide legal protection to non-lichenized fungal species and 16 to lichenized fungi. The number of protected taxa varied widely, from three to 420 species for non-lichenized fungi and from one to 472 species for lichenized fungi (Table 1; Fig. 2A, C). A small subset of countries accounted for a disproportionate share of protected taxa: Croatia (420), Finland (247), and Poland (114) together accounted for 88.3% of protected non-lichenized fungi, while Finland (472), Poland (199), and Luxembourg (117) accounted for 93.4% of protected lichens.

Hierarchical clustering based on Sørensen dissimilarity revealed distinct geographic clustering among countries that legally protect fungi (Fig. 2B) and lichens (Fig. 2D). The three Baltic states (Estonia, Latvia, and Lithuania) clustered together in both groups, while Hungary, Poland, and Slovakia clustered together for fungi. Mantel tests confirmed a significant positive correlation between Sørensen dissimilarity and geographic distance between country centroids for both fungi (Spearman *r* = 0.58, *p* = 0.001) and lichens (*r* = 0.45, *p* = 0.001), showing that neighboring countries tend to protect similar species. Nestedness analysis showed that protection lists for non-lichenized fungi were significantly nested (NODF = 20.7, *z* = 5.76, *p* = 0.001), i.e., countries with fewer protected fungi tended to protect a subset of widely recognized priority species. Conversely, lichen protection lists were not significantly nested (*p* > 0.05).

**Figure 3.**
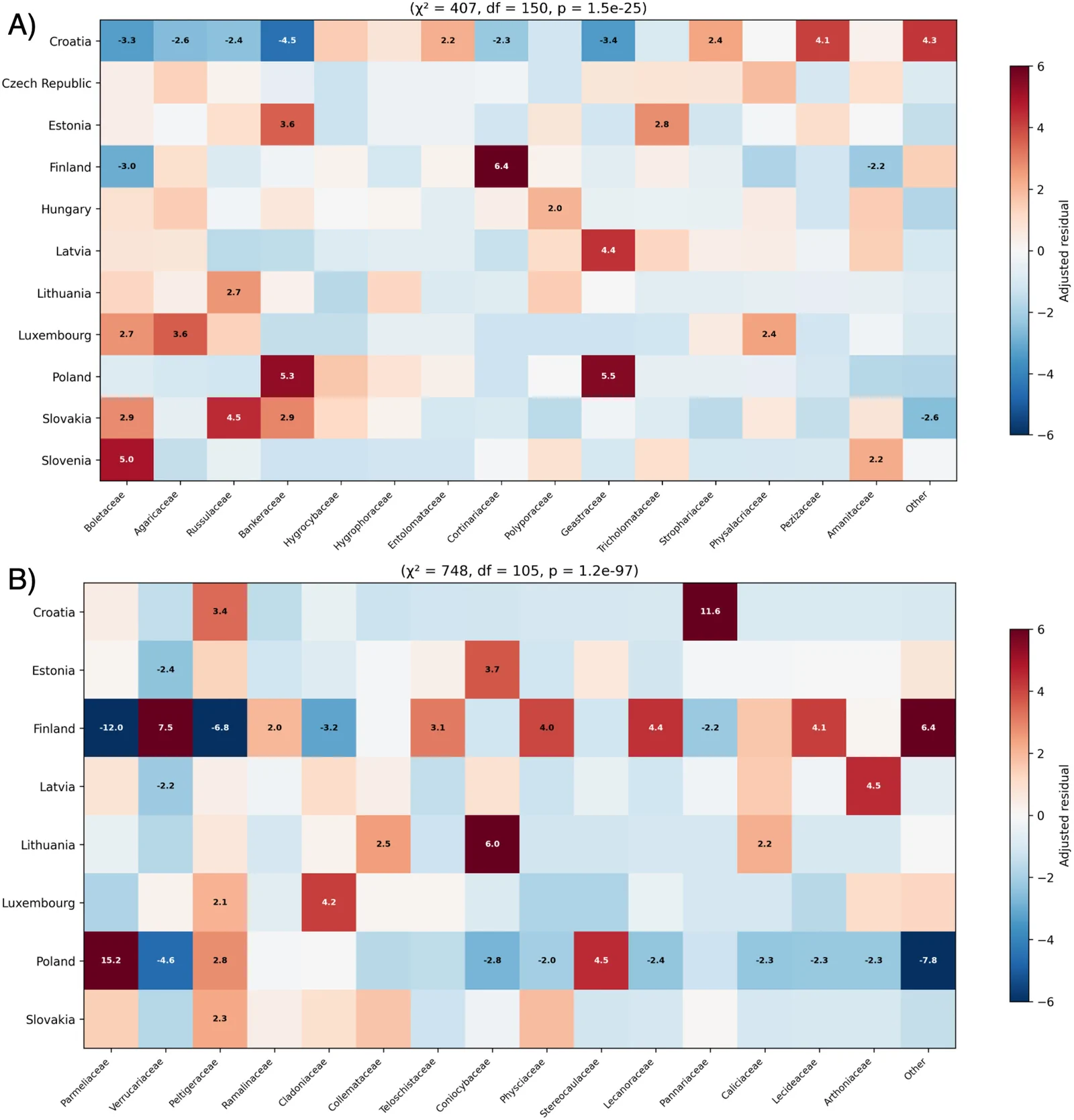
Adjusted standardized residuals from chi-square tests of independence for the taxonomic family composition of national protection lists across EU member states. Only countries with more than 20 protected taxa in the respective group were included. Values are shown only for significant cells (|adjusted residual| ≥ 2.0); positive values (red) indicate overrepresentation, whereas negative values (blue) indicate underrepresentation. **A**. Non-lichenized fungi. **B**. Lichenized fungi.

Countries differed significantly in their family coverage of protected taxa (non-lichenized fungi: χ^2^ = 407, df = 150, *p* < 0.001; lichens: χ^2^ = 748, df = 105, *p* < 0.001). Among fungi, Finland overrepresented *Cortinariaceae* (adjusted residual = +6.4), Poland overrepresented *Bankeraceae* (+5.3) and *Geastraceae* (+5.5), and Slovenia and Slovakia emphasized *Boletaceae* (+5.0 and +2.9, respectively). Among lichens, Poland’s list was dominated by *Parmeliaceae* (+15.2), while Finland overrepresented *Verrucariaceae* (+7.5) and other crustose families, Croatia overrepresented *Pannariaceae* (+11.6), and Lithuania overrepresented *Coniocybaceae* (+6.0) (Fig. 3).

**Figure 4.**
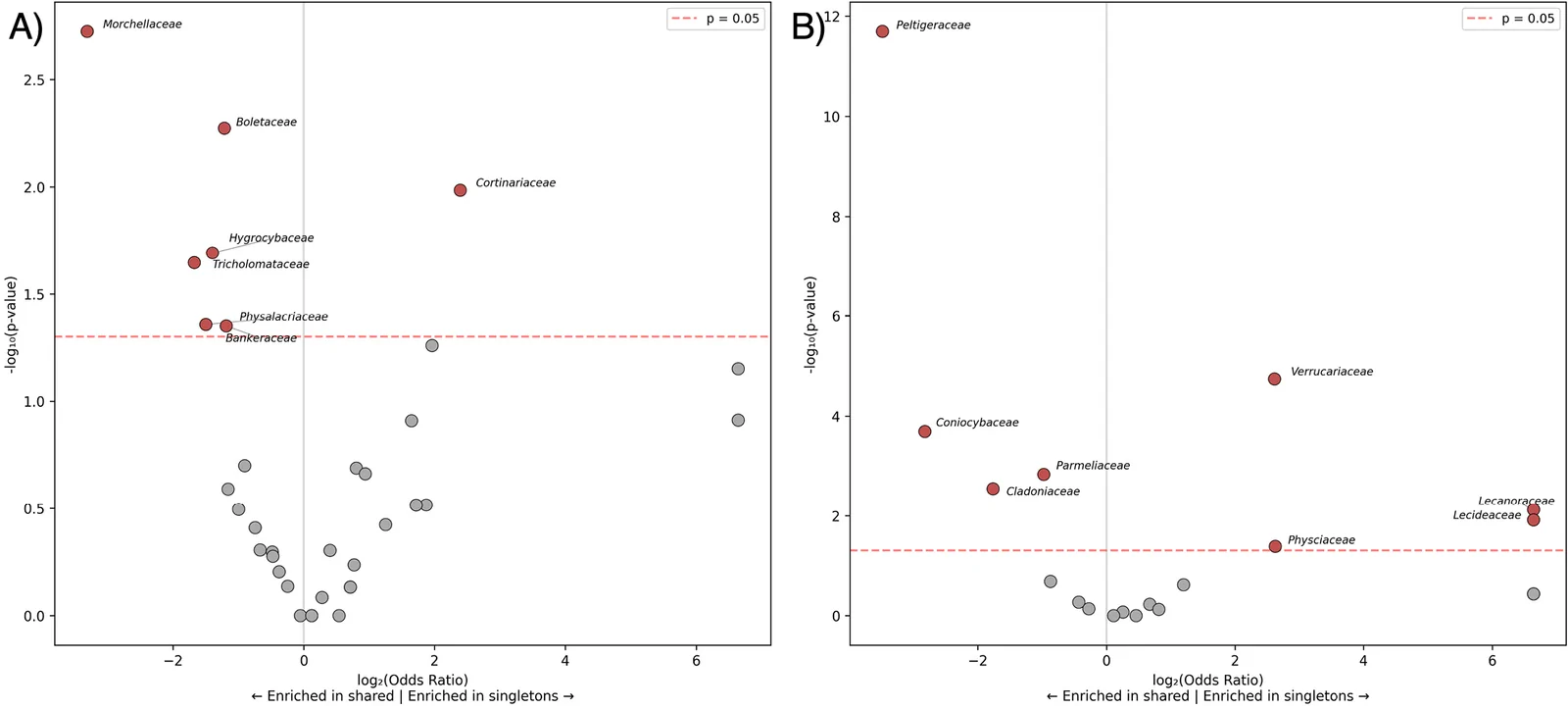
Family enrichment analysis comparing singleton taxa (protected in only one country) with shared taxa (protected in two or more countries). Each point represents a taxonomic family with at least eight taxa; significant families (Fisher’s exact test, *p* < 0.05) are shown in red and labeled. The x-axis shows log_2_-transformed odds ratios, with positive values indicating enrichment among singletons and negative values indicating enrichment among shared taxa. **A**. Non-lichenized fungi. **B**. Lichenized fungi.

Of the 884 protected non-lichenized fungal taxa, 649 (73.4%) are singletons. The singleton rate is higher for lichens: 630 of 787 taxa (80.1%). Countries with the largest lists contribute disproportionately: Croatia (279 singletons, 66.4% of its list) and Finland (177, 71.7%) together account for 70.3% of all fungal singletons. Finland alone contributes 64.9% of all lichen singleton taxa (409 singletons, 86.7% of its list). Singletons are significantly less likely to be assessed by the IUCN than shared taxa: among fungi, only 8.6% of singletons have global IUCN assessments, compared with 28.9% of shared taxa (Fisher’s exact test, OR = 0.23, *p* < 0.001). Family composition also differs between singletons and shared taxa: among fungi, *Cortinariaceae* are enriched among singletons (OR = 5.3, *p* = 0.010), whereas *Morchellaceae* are most enriched among shared taxa (OR = 0.10, *p* = 0.002). Among lichens, *Lecanoraceae* (OR = undefined, *p* = 0.008) are most enriched among singletons, whereas *Peltigeraceae* (OR = 0.09, *p* < 0.001) are most enriched among shared taxa (Fig. 4).

Among protected non-lichenized fungi, 124 are included on the global IUCN Red List: four are Data Deficient, 50 are Least Concern, 22 are Near Threatened, 43 are Vulnerable, and five are Endangered. Of the 33 priority threatened fungi in Europe (Dahlberg and Croneborg 2006), 29 are protected in at least one EU country. Only five protected lichenized fungi appear on the global IUCN Red List (four Least Concern and one Near Threatened). Five lichen taxa listed above the species level include species on the IUCN Red List; however, these are predominantly taxa native to North and South America.

## Discussion

The consolidated lists of legally protected non-lichenized and lichenized fungal taxa presented here represent the first EU-wide synthesis of national fungal protection data. By integrating information dispersed across national legal systems and multiple languages and standardizing taxonomic nomenclature, this compilation improves the transparency of the legal status of fungi in Europe. Increasing the visibility of species protection laws is particularly important, as accidental non-compliance (i.e., violations due to unawareness) is a major factor limiting the effectiveness of conservation laws (Jokinen et al. 2018).

Multivariate analyses show that national protection lists are not fully independent and instead follow geographic patterns: neighboring countries tend to protect more similar species. The nestedness of non-lichenized fungal lists suggests a partial consensus on “core” conservation-priority species that are consistently protected across several EU member states. The absence of nestedness among lichen lists, together with the taxonomic uniqueness of protection lists in countries with extensive lichen protection (e.g., Finland and Poland), suggests that international consensus on lichen conservation priorities is more limited. This may seem unintuitive, given that the only fungal taxon protected at the EU level is the lichenized *Cladonia* sect. *Cladina*. However, the European consensus on the protection of non-lichenized fungi may be explained by years of fungal conservation advocacy, primarily focused on non-lichenized taxa (e.g., Dahlberg and Croneborg 2006; Floccia et al. 2024).

While the consolidated lists presented offer an immediate reference, they also underscore the need for an official, centralized EU database of protected species. In addition to increasing transparency and availability, an EU-level database could be regularly updated to reflect changes to protected species lists across member states. The European Nature Information System (EUNIS; eunis.eea.europa.eu) already includes a module for searching “information about species in Europe, particularly species mentioned in legal texts.” However, fungi and lichens are poorly represented, and the few included taxa have largely incomplete profiles. Even for groups better represented in conservation law (e.g., animals), only international legal documents are referenced, and no information on their legal status in individual member states is provided. Improving this infrastructure by requesting that member states report up-to-date information on nationally protected species and the legal documents that regulate their protection and by including this information in the system would be a relatively straightforward improvement at negligible financial or political cost.

A centralized database of protected species in the EU would also help standardize taxonomy across the legislation of different member states. One difficulty in consolidating national lists of protected fungi was the use of taxonomic synonyms, outdated species names, and occasional spelling inconsistencies. Such variation creates barriers to enforcement and monitoring, particularly for stakeholders without specialist mycological expertise. A centralized EU system could dynamically link protected taxa to currently accepted names, with synonyms used in legislation included as additional information in each species profile, as in the consolidated lists presented in this work. A complementary solution to avoid ambiguity would be integration with a taxonomic reference system, such as the Global Biodiversity Information Facility (GBIF) Backbone Taxonomy, to ensure naming consistency and introduce persistent taxon identifiers (GBIF taxonKey) that facilitate tracking taxa across taxonomic revisions (GBIF Secretariat 2023).

Examining the taxa protected across EU countries reveals a systematic bias toward fungi that form conspicuous sporocarps. Among non-lichenized fungi, the most represented families (*Boletaceae*, *Agaricaceae*, and *Russulaceae*) typically produce large, mushroom-like sporocarps, and many species are culturally familiar through culinary use (Schulp et al. 2014). In contrast, taxa producing less conspicuous or hypogeous sporocarps are protected in fewer countries, and microfungi are scarcely represented. While less relevant to regulating individual behavior, including microfungi and other inconspicuous organisms in protected species lists can create valuable legal tools for regulating the industrial sourcing of microorganisms and defining which taxa are considered in environmental impact assessments and ecological monitoring in protected areas (Arya and Shinde 2022).

Singleton analysis shows that most fungi are protected in only one country and would lose their only legal protection in the EU if that country amended its legislation. Although national protection lists are based on different criteria and singleton status alone does not imply a need for EU- or global-level protection, many singletons represent cryptic (e.g., *Verrucariaceae*) or taxonomically challenging (e.g., *Cortinariaceae*) groups that are underrepresented in broader conservation assessments. Consequently, any genuinely rare and declining singleton taxa remain poorly represented within international conservation frameworks. Prioritizing IUCN assessments and population surveys for singleton taxa, especially in families in which singletons are overrepresented, would help identify species that require broader protection measures vs. those for which current single-country protection is sufficient.

National laws listing protected fungi are updated at different intervals across EU member states. In some countries, these laws have not been updated for more than a decade since their introduction (e.g., Slovenia 2011; Poland 2014). During such periods, conservation status and threat levels can change substantially, leading to misalignments between legal and ecological realities. A potential opportunity to standardize revisions to protected species lists in EU member states is the recently adopted Nature Restoration Regulation (NRL; European Union 2024). Among its provisions, the NRL requires member states to conduct detailed environmental monitoring, with results reported in 6-year cycles. Integrating fungal diversity into these monitoring frameworks would be a cost-effective way to collect data on populations of protected taxa (Dahlberg and Mueller 2011) and would improve the overall resolution of habitat-condition monitoring (Jönsson et al. 2017; Grzesiak et al. 2026).

Global IUCN Red List coverage of fungi has grown substantially in recent years, with the first global fungal Red List assessments published only in 2003 (Mueller et al. 2022). While many European non-lichenized fungal taxa are now included on the global IUCN Red List (IUCN 2026), lichenized fungi are largely absent. Searching the Red List using lichen genus names yields several species with conservation assessments; however, these are predominantly taxa native to North and South America, indicating a geographic bias in lichen conservation assessments. Although numerous European lichen taxa are considered conservation priorities nationally (787 species protected across EU member states), they remain unevaluated on a broader scale. This misalignment is particularly concerning, given that 80.1% of protected lichen taxa are singletons. Single-country dependency and the lack of global assessments make European lichens especially vulnerable to policy changes at the national level. A systematic IUCN assessment of European lichen taxa is necessary to provide a more accurate global picture of their population status.

The effectiveness of legal species protection varies across groups of organisms. The best conservation outcomes are observed in charismatic animal taxa. One such group is birds: populations of bird species listed in Annex I of the Birds Directive have improved relative to those of unlisted species, providing evidence that international species protection instruments can be effective (Donald et al. 2007; European Union 2009). However, protected insect and plant species do not perform equally well; this is largely due to the limited proportion of conservation programs and funding directed toward them (Mammides 2019; Mammola et al. 2020). Unfortunately, fungi and lichens are at the end of the popularity gradient that guides the allocation of conservation project funds. Moreover, sparse distribution data and limited monitoring and enforcement constrain the benefits of legal protection for fungal species. Nevertheless, the value of fungal and lichen species protection should not be underestimated.

Legal species protection is often viewed as an instrument for governing individual behavior. For fungi, this can entail protecting popular edible species to limit harvesting, although evidence that harvesting itself threatens fungal populations is weak: long-term monitoring has shown no effect of sustained picking on subsequent fruiting or fungal species richness (Egli et al. 2006). However, listing a taxon also creates legal instruments that may direct the actions of organizations and public authorities: protected species become relevant to permitting procedures, environmental impact assessments, pre-investment surveys, and the design of monitoring in protected areas (Arya and Shinde 2022). When permits for actions affecting protected species are issued, they leave a record documenting the applicant, the justifications provided, and the deciding authority—information that would be missing for unlisted species. Legal protection therefore introduces systemic accountability and generates an information trail valuable to conservation practice.

Habitat protection supports fungal and lichen conservation but does not functionally replace the aforementioned contributions of species protection. This approach operates by designating protected sites or habitat types and establishing associated management obligations. Importantly, habitat-based approaches can also preserve “invisible” fungal diversity, including cryptic and undescribed taxa (Blaalid and Davey 2022). Given sufficient time, habitat protection can also allow threatened fungi to rebuild their populations; an example of this was observed in recent years for *Amylocystis lapponica* in Estonia (Runnel et al. 2020). However, species listing remains the mechanism by which a taxon enters permitting and assessment procedures, whereas habitat designation governs local land use regardless of which particular species are present. Notably, although no non-lichenized fungus is protected under EU law, the Habitats Directive protects natural habitat types that host substantial fungal diversity (e.g., Grzesiak et al. 2026), so European fungi already benefit from habitat-level instruments, albeit incidentally. Making this benefit deliberate rather than accidental, i.e., listing fungal species alongside animals and plants and considering them when habitats are designated, would strengthen both instruments: species listing and habitat designation.

## Conclusion

This work shows that legal protection for fungal species exists in the EU but is fragmented across national legislation, with limited international coordination. More than three-quarters of protected fungi and lichens are protected in only one country. The lack of consolidated, accessible information on fungal species protected across EU member states hampers cross-border cooperation on fungal conservation and may limit the effectiveness of these laws. While fungi would certainly benefit from being incorporated into international instruments for species protection (e.g., the Bern Convention), increasing the visibility of current national regulations is a significant step toward improving fungal conservation in the EU and beyond.

## Additional information

### Artificial Intelligence (AI) use

The author accepts full responsibility for the content of the manuscript, including the disclosure of any use of AI.

Regarding the use of AI in the preparation of this manuscript, the author declare the following: Claude, Grammarly.

**Used for**: Language, style and writing, Software and automation.

Grammarly was used to improve the language and style of the manuscript. Claude was used to facilitate the preparation of Python scripts.

### Adherence to national and international regulations

All the fungal strains used in this study have been legally obtained, respecting the Convention on Biological Diversity (Rio Convention).

### Author ORCIDs

D. Janowski https://orcid.org/0000-0002-9929-6969

### Data availability

All of the data that support the findings of this study are available in the main text or Supplementary Information.

## Supplementary materials

Supplementary material 1 EU Legal protection list of fungi and lichens https://doi.org/10.3897/imafungus.17.202399.suppl1 Data type xlsx Explanation note EU_Legal_Protection_List_Fungi_Lichens.xlsx (A1: protected non-lichenized fungal taxa; A2: protected lichenized fungal taxa). https://binary.pensoft.net/file/1753532This dataset is made available under the Open Database License (http://opendatacommons.org/licenses/odbl/1.0/). The Open Database License (ODbL) is a license agreement intended to allow users to freely share, modify, and use this Dataset while maintaining this same freedom for others, provided that the original source and author(s) are credited. Daniel Janowski
